# 

**Published:** 1781-10

**Authors:** 


### PROMOTIONS.

Dr. James Harvey to be a Candidate, and Dr. William Keir and Dr. William Paine to be Licentiates of the College of Physicians, London.

*October* 23. Mr. Alexander Lindsay to be surgeon to the Royal Irish regiment of artillery.

---

### DEATHS.

Lately, at Dublin, Mr. Alexander Cunningham, senior surgeon to the Meath Hospital in that city. He had just finished the operation of lithotomy on a patient in the hospital, and extracted a stone of unusual bulk, when he fell suddenly dead at the feet of his friends, who were complimenting him with great eagerness on his dexterity.

*Sept.* 1. At Old Melrose, Roxburghshire; Dr. John Caverhill, member of the College of Physicians, London, F. R. S. and author of the following works: "Observations on the "Knowledge of the Ancients in the East In-"dies." Phil. Transf. vol. lvii. "A Treatise "on the Cause and Cure of the Gout." 8vo. London, 1769. "Experiments on the Causes "of

"of Heat in living Animals." 8vo. ib. 1770.

"A Dissertation on Nervous Ganglions and

"Nervous Plexus." 8vo. ibid. 1772.

*Sept.* 10th. Suddenly, of the gout in his stomach, Mr. William Cooper, surgeon to the Infirmary at Shrewsbury.——17th. At South End, near Dagenham, Essex, Dr. John Letch, F. R. and A. S.——18th. At Llanfylling, Montgomeryshire, Mr. Davies, surgeon and apothecary.—28th. At Holland, in Lancashire, Richard Meyrick, M. D.

*October* 9th. At Nottingham, Mr. Edward Merry, apothecary.——10th. In Ludgate-street, London, Mr. Alexander Dalmahoy, member of the Company of Apothecaries.——12th. In Charles-street, St. James's Square, Dr. Thomas Brooke, fellow of the Royal College of Physicians, and physician to St. Luke's Hospital.

---

### SECTION IV.

### MONTHLY CATALOGUE.

1. THE Medical Pocket Book. Containing a short but plain account of the symptoms, causes, and methods of cure of the diseases incident to the human body; including such

ſuch as require ſurgical treatment: together with the virtues and doſes of medicinal compoſitions and ſimples. Extracted from the beſt authors, and digeſted into alphabetical order. By *John Elliott*, M. D. 12mo. *Johnſon*, London, 1781. 136 pages. 2s.

This compendium will be uſeful to medical practitioners in general, either by aſſiſting their memory in matters already known, or by affording information to thoſe who have not leiſure or opportunity to conſult larger works. As a ſpecimen of the author's manner, we ſhall extract his account of the ague:

"Symptoms. The fit begins with cold ſhiverings; a ſmall quick pulſe; pain in the back and head; nauſea. To theſe ſucceed great heat and fever, which terminate in ſweats. The urine during the fit, pale, clear, and without ſediment; but in the interval, turbid, with a copious ſediment of a reddiſh colour.

In the *Quotidian ague* the fit returns once in a day.

In the *Tertian*, every other day.

In the *Quartan*, the intermiſſion is of two whole days.

Treatment. Firſt give an emetic, and afterwards a gentle cathartic. If the intermiſſions are not not regular, faline febrifuges fhould be admi-niftered till that objection is removed; then give the bark, in fubftance ʒj. every two hours during the intermiffion, adding tinct. theb. or other aftringent if it runs off by ftool. If the ftomach will not bear the powder, give it in decoction, infufion, or the extract in pills. Pulv. fl. chamæm. chalybs. rad. ferp. virg. elix. vitr. acid. or the like, may be added according to circumftances. The repetitions may be lefs frequent after the fit has been miffed once or twice. Vitr. cærul. gr. ff. diffolved in ℥j. of proof fpirit, and given before the fit, has fucceeded in fome defperate cafes."

2. A Philofophical and Experimental Enquiry into the firft and general principles of animal and vegetable life: likewife into atmofpherical air, with a minute inveftigation of the different fecondary principles attendant upon each: viz. animal heat, fanguification, animal moifture, age, temperament, &c. &c. &c. with a refutation of Dr. Prieftley's doctrine of air; proving by experiment, that the breathing of animals, putrefaction, &c. do not phlogifticate, but dephlogifticate the air; and that the office of that effential organ, the lungs, is not to difcharge phlogifton to the air, but to receive it from from the air. By *Robert Harrington*, of the Corporation of Surgeons, London. 8vo. Cadell, London, 1781. 402 pages.

3. Prefens de Flore à la Nation Françoife, pour les alimens, les medicamens, l'ornement, l'art veterinaire, les arts & metiers; ou traité hiftorique des plantes qui fe trouvent naturellement dans les differentes provinces du Royaume, rangées fuivant le fyfteme de M. le Chevalier de Linné, avec tous les details qui les concernent: par *M. Buchoz*, medecin de Monfieur, &c. *i. e.* Prefents of Flora to the French nation, for aliment, medicine, ornament, the treatment of cattle, arts, and trade; or an hiftorical treatife of the plants that are found in the different provinces of the kingdom, arranged according to the Linnæan fyftem, with all the particulars that relate to them. By M. *Buchoz*, phyfician to Monfieur, &c. 4to. vol. i. Paris, 1780. 208 pages.

4. Obfervations fur la nature, les caufes, et le traitement de la fievre lente ou hecticque. Par *M. Fournier*, Docteur en medecine de la faculté de Montpellier, de la Société Royale des Sciences, medecin des Etats Generaux du Duché du Bourgogne. *i. e.* Obfervations on the nature, caufes, causes and treatment of the hectic or slow fever. By *M. Fournier*, M. D. of the faculty of Montpellier, of the Royal Society of Sciences, physician to the States General of the Dutchy of Burgundy, &c. 8vo. Dijon, 1781. 215 pages.

The author of this work professes to give it as the result of sixty years experience. It is divided into two parts, each of which is subdivided into chapters. In the first chapter of the first part he points out the nature, diagnosis, and prognosis of the hectic fever, of which he distinguishes two species. One of these, which he calls idiopathic, he supposes to be owing to a general depravity of the fluids. The other he stiles secondary, or symptomatic, because it accompanies all internal, and sometimes even external suppurations. The proximate cause of both these species, he ascribes to an obstruction of what he calls *vaisseaux neuro-lymphatiques*, neuro-lymphatic vessels. The explanation of this singular theory employs his second chapter, in which we meet with a great deal of unintelligible matter. In the third he describes the symptoms of the three stages of hectic fever.

The second part of the work is allotted to the treatment of this fever. M. Fournier does not undertake to treat of every species, but confines himſelf chiefly to the pulmonary hectic, to that which is produced by corroſive poiſonous ſubſtances taken internally, and laſtly to the venereal hectic.

He deſcribes the effects of ſeveral poiſonous medicines adminiſtered by quacks. He makes mention of an empiric, named Troublot, who uſed to cure intermittents by a pill, the baſis of which was arſenic. Almoſt all his patients died hectic a few months after they had been under his care. M. Fournier quotes inſtances of a ſimilar effect from corroſive ſublimate given in the venereal diſeaſe.

5. Catechiſme ſur les morts apparentes, dites aſphyxies, ou inſtruction ſur les manieres de combattre les differentes eſpeces de morts apparentes, par demandes et par reſponſes. Par *M. Gardane*, Docteur-regent, &c. *i. e.* Catechiſm on apparent deaths, commonly called aſphyxies, or an account of the methods of treatment in the different ſpecies of apparent death; by queſtion and anſwer. By *M. Gardane*, Doctor-regent of the Faculty of Phyſic at Paris, Cenſor Royal and member of the Academies of Sciences at Montpellier, Nancy, and Marſeilles. 8vo. Paris, 1781. 116 pages.

This performance, which is written in a popular pular ſtyle, is printed at the expence of government.

6. Introduzione alla medicina pratica, &c. *i. e.* Introduction to the practice of Phyſic, by *P. A. Gallo*, M. D. 8vo. Vezecil, 1779.

In a preliminary diſcourſe the author offers ſome remarks on the origin of diſeaſes, the utility of phyſic, and the education, &c. of phyſicians. The work itſelf is divided into four chapters. In the firſt the author treats of the ſolids and fluids of the human body, and of the difference of age, ſex, &c. after which he points out the miſtakes of phyſicians in inflammatory, and what he calls lymphatic fevers, and then ſpeaks of the nature and treatment of epidemical diſeaſes.

In the ſecond chapter Dr. Gallo after treating of worm fevers, and their remedies, ſpeaks of hectic fever. He aſcribes the latter of theſe to a vitiated ſtate of the lymph, or to obſtructions of the meſentery or worms, and is of opinion, that the means of cure are chiefly to be ſought for in regimen.

The third chapter is allotted to the diſeaſes that are occaſioned by ſome affection of the ſolids, by diverſity of temperament, the paſſions of the mind, the uſe of mercury, different

ſpecies of gout and inflammation. In the fourth and laſt chapter, Dr. Gallo treats of diſeaſes, the cauſe of which is ſuppoſed to exiſt in the fluids.

7. Diſcours philoſophique ſur les trois principes animal, vegetal, & mineral, ou la clef du ſanctuaire philoſophique. *i. e.* A Philoſophical diſcourſe on the three principles, animal, vegetable, and mineral; or a key to the philoſophical ſanctuary. By *Sabine Stuart de Chevalier*. 12mo. Paris, 1781. 2 vols.

An alchemiſtical rhapſody.

8. Diſſertatio Inauguralis de Vaſis plantarum ſpeciatim radicem herbamque adeuntibus. Auctore *Joanne Henrico Daniel Moldenhawer*, Regiomonte Boruſſo, 4to. Trajecti ad Viadrum, 1779. 85 pages.

9. Tal om Sjukligheten e faelt. *i. e.* A Diſcourſe on the diſeaſes of the army, during the campaigns in Pomerania from 1752 to 1762, read before the Royal Academy of Sciences at Stockholm, on quitting the office of Preſident of that Society, July 21, 1779. By *P. Zetzell*, M. D. member of the College of Phyſicians at Stockholm, and phyſician to the Swediſh army. 4to. Stockholm, 1779.

This

This work appears to be the refult of accurate and extenfive obfervation. The author defcribes the various fituations of the army, and endeavours to afcertain the influence of provifions, cloathing, weather, and the other caufes that concur in producing military difeafes. He obferves, that putrid and intermittent fevers, and dyfentery, were the moft prevailing difeafes. At the beginning of 1759 the troops were attacked in the proportion of one in ten with putrid fever, and of one in thirty with dyfentery.

In fpeaking of cloathing he obferves, that too tight cloathing fometimes occafions fudden death. He has feen four foldiers die fuddenly from this caufe, and three others who were faved with difficulty. From his journal it appears, that the proportion of fick varied from 1 in 20, to 3 in 11, fo that in an army of 30,000 men there will upon an average be 2500 fick. He recommends fmall hofpitals.

Dr. Zetzell obferves, that the three firft years of a war are much more fatal to troops than the following ones, and that in Northern climates the number of fick is always greater in winter and fpring than in fummer and autumn. But there is no month—he adds— in which in camps there are not cafes of chronic rheumatifm, apoplexy, plexy, palſy, epilepſy, phthiſis, inflammatory colic, hypochondriaſis, tænia, lues, &c. He has obſerved, that intermittents are more rife when the cold as well as the moiſture is moderate; that too moiſt an atmoſphere produces putrid fevers and dyſentery; that periodical pains of the head and face are moſt prevalent when the air is moderately cold and damp, and that long continued rains occaſion itch, catarrhs, dropſical complaints, aſthma, epilepſy, and hypochondriaſis.

The author endeavours to aſcertain the parts of the body that are the moſt frequently wounded in battle. He is convinced that in a battle between two armies, compoſed of infantry, the upper half of the body is the moſt in danger, and that for two ſhot in the belly, three or four will be wounded in the neck or breaſt, ſeven in the head, ten in the arms and hands, four in the hips, five in the legs, one in the knee, and two in the feet.

10. Cours complet d'Agriculture, theorique, pratique, economique, et de medecine rurale et veterinaire, ſuivi d'une methode pour etudier l'agriculture par principes; ou Dictionnaire Univerſel d'Agriculture, par une Société d'Agriculteurs, et redigé par M. l'Abbé *Rozier*, Prieur-Com-

Commendataire de Nanteuil, membre de plusieurs Academies, &c. *i. e.* A Complete Course of Agriculture, theoretical, practical, and œconomical; and of rural and veterinary physic; to which is added a rational method of studying agriculture; or an Universal Dictionary of Agriculture by a Society of Farmers, arranged by the Abbé *Rozier*, Prior of Nanteuil, and member of several academies. 4to. Paris, 1781. vol. i. 704 pages.

Botany, chemistry, the diseases of cattle, and in short every thing that relates to rural œconomy, is very minutely treated of in this work, which will probably be very voluminous. This first volume includes only part of the letter A. The word *Abeille (bee)* alone employs 167 pages.

11. Tentamen Medicum Inaugurale de Viribus Corticis Peruviani, ejusque usu in morbis non febrilibus. Auctore *Joanne Cooke*, Anglo, Reg. Societ. Medic. Edinburg. Socio. 4to. Lugduni Batavorum, 1781. p. 23.

12. Memoire Clinique sur les maladies veneriennes. *i. e.* A Clinical essay on venereal complaints. 8vo. Paris, 1780. 308 pages.

13. Della morte apparente degli annegati. *i. e.* Of the apparent death of drowned persons. By

*Anthony*

*Anthony Joseph Testa.* 8vo. Florence, 1780, 200 pages.

The author allows that in certain cafes the water penetrates into the lungs of drowned perfons; that refpiration is ftopt, and the blood collected in the veffels of the head and thorax; but he denies that death can be brought on by thefe caufes, and ferioufly afcribes it to the phlogifton retained in the lungs, and exerting its effects on the nervous fyftem. He does not pretend to fay how this phlogifton acts, but he fuppofes that a circulation is kept up, though weakly. He confiders the froth that iffues from the mouth of drowned perfons, and others who die fuffocated, as being nothing more than air thrown off from the lungs, and mixed with the mucus of the mouth and noftrils. His methods of recovery differ not from thofe in general ufe.

14. Differtatio Inauguralis Pharmacologica de Sternutatoriis. Auctore *Francifco Alexandro Mangin.* 4to. Nancy, 1781.

The author diftinguifhes thofe medicines which excite fneezing from *errhines* or fuch as produce only an increafed fecretion of mucus in the noftrils. He gives a lift of each.

